# Fluorescent Nanobiosensors for Sensing Glucose

**DOI:** 10.3390/s18051440

**Published:** 2018-05-05

**Authors:** Longyi Chen, Eugene Hwang, Jin Zhang

**Affiliations:** 1 Department of Chemical and Biochemical Engineering, University of Western Ontario, 1151 Richmond St., London, ON N6A 5B9, Canada; lchen449@uwo.ca; 2 Biomedical Engineering Graduate Program, University of Western Ontario, 1151 Richmond St., London, ON N6A 5B9, Canada; ehwang8@uwo.ca

**Keywords:** glucose sensing, fluorescent nanomaterials, fluorescent nanobiosensors, sensitivity and selectivity

## Abstract

Glucose sensing in diabetes diagnosis and therapy is of great importance due to the prevalence of diabetes in the world. Furthermore, glucose sensing is also critical in the food and drug industries. Sensing glucose has been accomplished through various strategies, such as electrochemical or optical methods. Novel transducers made with nanomaterials that integrate fluorescent techniques have allowed for the development of advanced glucose sensors with superior sensitivity and convenience. In this review, glucose sensing by fluorescent nanobiosensor systems is discussed. Firstly, typical fluorescence emitting/interacting nanomaterials utilized in various glucose assays are discussed. Secondly, strategies for integrating fluorescent nanomaterials and biological sensing elements are reviewed and discussed. In summary, this review highlights the applicability of fluorescent nanomaterials, which makes them ideal for glucose sensing. Insight on the future direction of fluorescent nanobiosensor systems is also provided.

## 1. Introduction

Historically, glucose has been analyzed by colorimetric reaction methods. The most widely used method is Miller’s colorimetric method [1]. Glucose solution has no absorbance and no fluorescence in the visible range. Therefore, most of the analysis methods in early days were dependent on the glucose chromogenic reaction. For example, in the Miller method, the glucose carbonyl group (C = O) is oxidized and, at the same time, 3, 5-dinitrosalicylic acid (DNS) is reduced to 3-amino, 5-nitrosalicylic acid under alkaline conditions with colored products. The glucose content is related to the colorimetric products, which could be quantified by UV-vis spectroscopy. 

Sensing glucose is very important in the food and pharmaceutical industries. In addition, glucose monitoring is especially critical for diabetes management. Diabetes is characterized by long-term hyperglycemia, and the monitoring of patients’ glucose levels is required for the management of the disease. The electrochemical method for sensing glucose is widely used among diabetes patients in the form of a blood glucose meter. However, the requirement for constant finger pricking in order to obtain a sample of blood for glucose sensing may cause discomfort and localized infections in patients. Therefore, patient-friendly, minimally invasive or non-invasive fluorescent detection methods have gained attention [2,3,4,5]. Fluorescent sensing has the following advantages [6,7,8,9]: (a) it is extremely sensitive, (b) it is minimally invasive or non-invasive, (c) fluorescence intensity and fluorescence lifetime can be utilized, (d) it can provide the structure and micro-environment of molecules, (e) the fluorescence resonance energy transfer (FRET) technique can be utilized. 

Nanotechnology has profoundly influenced the area of biosensors, particularly through their high sensitivity and selectivity, as well as the miniaturization of sensor devices. The development of nanotechnology allows materials to be prepared and fabricated in reduced dimensions at the nanoscale, forming nanostructures for high-throughput assays through the integration of microfluidics devices. In addition, the biological activity of the biological components is strengthened by using novel nanoscale materials and nanostructures. Nanomaterials and nanostructures have special chemical, physical, and biological properties compared to their bulk counterparts [10,11,12,13,14]. Fluorescent nanomaterials and nanostructures have distinct properties that can be taken advantage of in order to develop new nanostructured biosensors for glucose sensing [15,16,17,18,19,20]. Biosensors are sensors composed of or involved with a biological component [21,22,23,24]. Biological components have high sensitivity and selectivity to specific molecules; thus, the utilization of biological components can create highly accurate sensing devices for a variety of molecules including glucose, urea, and cholesterol.

**Figure 1:**
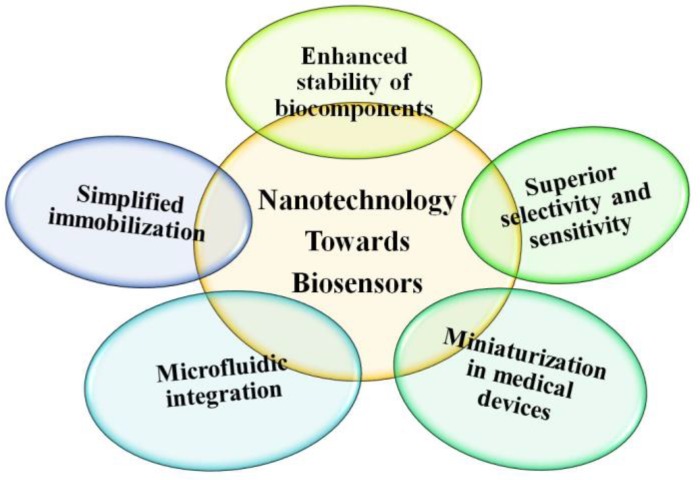
Nanotechnology contributions for the development of biosensors with commercial promises.

As shown in Figure 1, nanotechnology has had a profound influence in the biosensors area, the most obvious effect of which is the largely increased sensitivity and selectivity of nanobiosensors and the miniaturization of devices. The development of nanotechnology allows materials to present in reduced dimensions at the nanoscale, leading to the construction of nanostructures for high-throughput assays with the integration of microfluidics devices, and strengthening the biological activity of the biological components by using novel nanomaterials. Nanomaterials have special properties compared to their bulk counterparts. Special nanomaterials with size-dependent fluorescent properties provide unique platforms for developing new nanobiosensors for glucose sensing [15,16]. Biosensors are devices composed of or involved in a biological component which normally shows strong and specific interaction to an analyte, or to at target molecule such as glucose, urea, cholesterol, etc. [21]. 

Biosensors have been extensively applied in the food industry, pharmacy industry, environmental monitoring, and medical diagnostics, etc. Early biosensors used biocomponents as recognizing parts on the electrode surface. As nanotechnology has flourished, the conjugation of nanomaterials with biocomponents to form nanobiosensors has been widely studied and investigated. As nanomaterials and nanostructures are the equivalent size to biological systems, the nanobiosensors are able to provide unprecedented sensitivity and selectivity [25,26]. Some common biological receptors have been used in the measurement of glucose in fluorescent nanobiosensors, such as Concanavalin A (Con A), glucose oxidase, glucose dehydrogenase and hexokinase/glucokinase, glucose-binding proteins, etc. 

For fluorescent glucose nanobiosensors, fluorescence-emitting/interacting nanomaterials are used. In addition, biological components are involved in the glucose assay. Thus, the glucose-sensing system is recognized as a nanobiosensor. 

## 2. Fluorescence-Emitting/Interacting Nanomaterials

The most extensively researched fluorescence-emitting nanomaterials include fluorescent semiconductor quantum dots (QDs), dye-doped silica nanoparticles (DDSNs), lanthanide-doped nanomaterials, upconversion nanoparticles (UCNPs), and fluorescent gold/silver metal nanoclusters [27,28,29,30]. Some other nanomaterials such as fluorescence-interacting quenchers have been investigated in fluorescent assays as well. Plasmonic gold/silver nanoparticles are employed as fluorescent quenchers [31]. 

Carbon nanomaterials are also of great interest. Some carbon-based nanomaterials are fluorescent and others are fluorescent quenchers. Carbon nanomaterials such as graphene/graphene oxide, carbon dots, and carbon nanotubes have been studied for use in fluorescent glucose biosensing applications [32,33,34]. Graphene/graphene oxide nanomaterials are fluorescent quenchers due to their large π electron plane on the nanosheet plane, which can quench fluorescence through fluorescence resonance energy transfer (FRET). On the contrary, carbon dots and carbon nanotubes are promising strong fluorescent nanomaterials for biosensing applications. Carbon dots can be prepared through various facile methods and scaling up the production of carbon dots is not difficult. Carbon nanotubes emit fluorescence in the near-infrared range, which allows carbon nanotubes to be applied in bio-imaging. However, the cytotoxicity of carbon nanotubes needs to be further studied [35]. 

Some other fluorescent nanomaterials include metal oxide nanomaterials. As metal oxide nanostructures provide large effective surface areas for biomolecule immobilization, they are able to maintain better conformation, desired orientation, and high biological activity of immobilized biomolecules. ZnO nanostructured film is typically a weak fluorescent nanomaterial; however, it is often used as substrate for nanobiosensing. Other metal oxide nanostructures are often used in electrochemical biosensing due to their good electrical properties. However, metal oxide nanostructures are strong potential candidates for fluorescent glucose biosensing, and they could be further incorporated into such devices [36,37,38,39]. 

**Figure 2:**
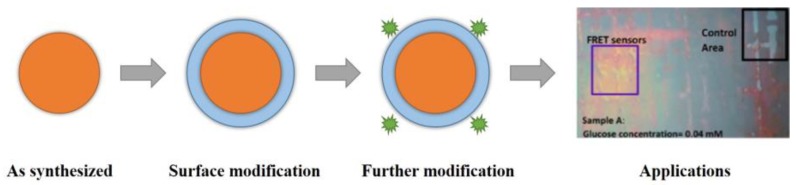
General steps involved in the application of functional nanomaterials.

Nanomaterials and nanostructures normally require surface functionalization to obtain desirable physiochemical properties for various applications. As shown in Figure 2, after the synthesis of a nanoparticle, surface coating is applied to form a core-shell nanoparticle. In addition, ligand exchange and functional groups are grafted or bioconjugated on the outer surface. The modification and functionalization of nanomaterials and nanostructures make them more water-soluble and biocompatible for further applications in biosensing systems. 

The aim of nanomaterial and nanostructure surface functionalization is to utilize them in catalysis, adsorption, drug delivery, bioimaging, nanobiosensor, and nanomedicine applications. Surface properties have a great influence on the nano-biointerface. Hydrophilic and hydrophobic surfaces can affect the interaction of nanomaterials with biomolecules and proteins adsorption. Related nanomaterials and nanostructures surface modification reviews [40,41,42,43,44,45] have demonstrated various chemical and physical modifications and bioconjugation methods. Various functional groups and applications of nanomaterials and nanostructures can be visualized with suitable surface modification and fabrications. 

### 2.1. Semiconductor Quantum Dots 

Quantum dots (QDs) are semiconductor nanoparticles with diameters in the range of 2–10 nm, containing roughly 200–10,000 atoms. They are characterized by size-dependent fluorescent properties, broad excitation range, narrow emission peak, large Stokes shift, ultrahigh brightness, high quantum yield, and photostability. The synthesis method of quantum dots is fairly simple and well established. The organic phase synthesis yields high-quality quantum dots. However, they are oil phase soluble, thus further surface modification is necessary to render their usage in biological sensing [46,47,48]. In addition, due to the cytotoxicity of the semiconductor element (Cd element), the bio-application of quantum dots is greatly hindered. A coating layer such as a silica shell, polymer shell, or carbon shell can be applied to the surface of the semiconductor quantum dots to improve their water solubility and biocompatibility [49,50,51,52]. Compared to traditional fluorescent dye, Green Fluorescent Proteins (GFP), or enhanced GFP, quantum dots have superior luminescent properties such as photostability to ambient environment, high quantum yield, and bright size-dependent excitation photoluminescence. These luminescent properties of semiconductor nanocrystals make them favorable as nanobiosensing probes [53,54]. 

### 2.2. Fluorescent Silica Nanoparticles 

Other fluorescent nanomaterials are dye-doped silica nanoparticles or polymer nanoparticles. Polymers nanoparticles can employ many different monomers and thus are highly varied. Dye-doped silica nanoparticles can be incorporated with fluorescent elements inside a polymerized matrix. Silica is a highly biocompatible material and the dye could be covalently conjugated or conjugated by physical doping into the silica matrix. Due to the silica protection of the doped dye, the photostability of the doped dye is greatly improved. Moreover, the silica matrix provides a very good surface platform for surface functionalization and the construction of various silica structures, including solid silica nanoparticles, mesoporous silica nanoparticles, hollow silica nanoparticles, core-shell silica nanoparticles, and Janus silica nanoparticles [55,56]. 

Silane could be adapted to functionalize the silica matrix with various functional groups such as amine groups (−NH_2_), carboxylic groups (−COOH), thiol groups (−SH), and epoxy groups (−CHOCH_2_). The most common silane monomer is Tetraethyl Orthosilicate (TEOS), and the most-used synthesis process is the sol-gel process. Mesoporous silica nanomaterials have a large specific surface area and the mesopores are ideal for loading fluorescent reporters. The mesopores could be gated by some target molecule. Silica or polymer nanoparticles represent useful tools for glucose sensing. 

### 2.3. Upconverting Nanoparticles

Upconversion nanoparticles (UCNPs) represent a large family of fluorescent nanomaterials. Upconverting nanomaterials have superior luminescent properties such as long luminescent lifetime, large anti-Stokes shift, narrow emission bands, and high photostability compared to quantum dots. Moreover, upconverting nanomaterials can be modified with proper surface coatings and functional groups in order to impart high biocompatibility and low toxicity, which would favor them as a new generation of fluorescent nanoprobes for biomedical applications [57,58,59,60,61]. Normally, near-infrared 980 nm excitation is used to excite upconverting nanomaterials to emit green, yellow, or red light in the visible range (400–700 nm). The emission peaks are sharp and tunable by adjusting the doping lanthanide elements and corresponding ratios. Commonly used doping elements include Er, Tm, and To, which enhance green, red, and yellow emissions, respectively [62,63,64]. 

### 2.4. Gold/Silver Nanoparticles/Nanoclusters 

Noble metal nanoparticles demonstrate the distinct surface plasmon resonance (SPR) phenomenon and are often utilized as fluorescence quenchers. Typical noble metal nanoparticles include gold, silver, and platinum nanoparticles. These plasmonic nanoparticles are widely used in biosensing applications. Interestingly, as the size of the noble metal nanoparticles decreases to the extent of several to tens of atoms, roughly smaller than 1 nm. These noble metal nanoclusters exhibit fluorescence properties that are tunable by controlling the nanocluster size or atom number [65,66,67,68]. 

Plasmonic gold metal nanoparticles are highly biocompatible as they are inert and stable in biological systems. The surface of gold atoms is especially susceptible to conjugation with thiol groups (−SH), which facilitate the surface functionalization of gold nanoparticles. These properties make gold nanoparticles very useful as fluorescent quenching nanobiomaterials for biosensing [69,70]. In the synthesis of most metal quantum clusters, proteins are always used to assist the formation and stabilization of nanoclusters. Nanoclusters with a particle size <1 nm have demonstrated special fluorescence properties. These nanoclusters are normally made of gold or silver, which are bio-inert and have good biocompatibility. The strong fluorescence comes from the discrete band gap due to their smaller size compared to gold/silver nanoparticles (2–50 nm). They have been widely investigated and applied in biosensing areas [71,72]. 

### 2.5. Fluorescent Carbon Nanomaterials 

Fluorescent carbon nanomaterials represent a broad family that includes carbon nanotubes, graphene oxide, graphene quantum dots, and carbon dots. Although the fluorescent mechanisms of some carbon nanomaterials are not yet fully understood, they have already been widely researched and applied in biosensing [73,74,75,76,77,78]. The main reason for their intense study is due to the inert nature of nanocarbon materials in biological systems, as well as their special fluorescent characteristics. With the proper surface coating of nanocarbon materials, their cytotoxicity is greatly lowered and more compatible to biological systems. In terms of fluorescence in carbon nanomaterials, graphene oxide has weak fluorescence, while graphene quantum dots, carbon dots, and carbon nanotubes have strong fluorescence. Multicolor graphene quantum dots and carbon dots have been widely studied. Graphene quantum dots (1–10 nm) are sometimes categorized as minimally sized graphene oxide (several hundred micrometers). Graphene quantum dots can be categorized as single-sheet carbon dots. Carbon dots are the strongest candidate for biosensing applications. The most distinguishable fluorescent characteristic of carbon dots is their excitation-dependent emission. As the excitation moves across from UV to near-infrared, the carbon dots emission shifts in correspondence with the excitation wavelength [79,80,81,82,83,84,85,86]. Some carbon dots have a stabilized emission peak even with a shifting excitation wavelength, while some carbon dots possess upconversion fluorescence when excited by near-infrared light [87,88,89]. 

### 2.6. Graphene Nanomaterials 

Some nanomaterials are fluorescence quenchers, like noble plasmonic gold/silver metal nanoparticles and graphene/graphene oxide. In a glucose analysis process, these fluorescent interacting nanomaterials could quench the fluorescent component through the non-radiative transfer of electronic excitation energy to the π electron system of graphene. When glucose is introduced, the fluorescence would be either turned on or turned off depending on the sensor design. Reduced graphene oxide (RGO) and graphene oxide (GO) are widely used in glucose sensing assays. Pure graphene is difficult to stabilize and functionalize. Reduced graphene oxide and graphene oxide are more extensively studied. RGO and GO have good water solubility and are also biocompatible, factors that favor their application in biosensing areas [90,91,92,93,94,95]. RGO/GO offer a large surface area and could be functionalized by various groups or nanomaterials. The original existing carboxylic groups, epoxy groups, and carbonyl groups on RGO/GO planes provide an anchor for further chemical modifications [96,97,98]. 

In summary, semiconductor quantum dots, dye-doped silica nanoparticles, and upconverting nanomaterials have been widely investigated and used in fluorescent glucose nanobiosensing. In addition, fluorescent quenching nanomaterials such as plasmonic gold/silver nanoparticles and graphene/graphene oxide nanomaterials have been used as fluorescence quenching components in glucose nanobiosensing assays. 

### 2.7. Table of Summary 

Here, a summary table of the above discussed fluorescence emitting/interacting nanomaterials is provided (Table 1). 

## 3. Application of Fluorescent Nanomaterials in Glucose Sensing

This section focuses on the application of the fluorescent nanomaterials and fluorescent quenching nanomaterials in glucose biosensing. The electrochemical glucose sensing method is far in development and some marketed products are already available and used in the clinical setting. However, some of the disadvantages of the electrochemical method include invasiveness and risk infection. The sensitivity and selectivity are prone to interference due to the complicated components in body fluid. As a result, the fluorescence nanobiosensor approach has gained more attention in recent years for its advantages of non-invasiveness as well as high sensitivity and selectivity. 

### 3.1. Fluorescenct Nanobiosensor Detection Principle

The principle of glucose fluorescent biosensor is based on converting the glucose signal to a correlated fluorescence signal. Three components are required [25]: (a) glucose-recognized biomolecules or molecules, (b) a signal transducer, and (c) a signal detector.

Fluorescent nanomaterials are good transducers for correlating glucose signals into fluorescent signals, either in peak intensity or peak shift and fluorescent lifetime. Fluorescence resonance energy transfer (FRET) is acknowledged as a sensitive and reliable analytical technique and is widely employed in fluorescence nanobiosensing systems. Nanomaterials can work as good vehicles and locating platforms for the glucose recognition in target cells or body fluid. By conjugation or physical adsorption on the nanomaterials, the biomolecules are greatly stabilized on the nanomaterials with higher biological activity.

The primary mechanisms used in glucose fluorescent nanobiosensing include glucose direct binding, competitive binding, and fluorescent dye release, which introduces corresponding changes in fluorescence or fluorescent lifetime. Other assays use glucose catalysis oxidation reaction products like hydrogen peroxide or gluconic acid (pH), which introduce fluorescence change. Some of the glucose oxidization processes are shown by the following equations. ATP represents adenosine triphosphate; ADP represents adenosine diphosphate. 

(1) $$Glucose+Concanavalin\;A⇋Concanavalin\;A^{*}$$

(2) $$Glucose+Glucose\;binding\;protein⇋Glucose\;binding\;protein^{*}$$

In Equations (1) and (2), the glucose-binding reactions are dynamic and reversible; the * mark represent the biomolecules bound with glucose.

(3) $$Glucose+Oxygen\overset{Glucose\;oxidase}{\to }Gluconic\;acid+Hydrogen\;peroxide$$

(4) $$Glucose+Oxygen\overset{Glucose\;dehydrogenase}{\to }Gluconic\;acid+Hydrogen\;peroxide$$

(5) $$Glucose+ATP\overset{Hexokinase/Glucokinase}{\to }Glucose\;6-phosphate+ADP$$

### 3.2. Glucose Recognition Biomolecules

Various groups of glucose recognition biomolecules have been investigated, including direct glucose binding by Concanavalin A, glucose-binding proteins, and glucose catalytic oxidization reactions by glucose oxidase, glucokinase, hexokinase, and glucose dehydrogenase. Concanavalin A (Con A) is a plant lectin protein extracted from Jack beans. The Con A tetramer molecule has four binding sites for glucose and is traditionally used in glucose competitive assays. Both the protein and competitors can be labeled by fluorophore. 

Another glucose-sensing biomolecule is the glucose-binding protein, which is present in living organisms like *Escherichia coli*. These binding proteins develop extremely high selectivity and sensitivity for their substrates. The binding constants of the proteins for their substrates are in the micromolar (µM) range. The sensitivity and selectivity of glucose-binding proteins make them ideal for biosensor development [99]. Their sensitivity and selectivity are tunable by recombinant protein engineering techniques [100,101], which offer a facile design to obtain desired glucose-binding protein for various areas of glucose concentration sensing.

Compared to other glucose-recognized biomolecules, glucose-binding proteins (GBP) are non-enzymatic glucose bioreceptors. GBP can bind glucose with high sensitivity and selectivity. The binding event can occur naturally and no by-products are produced. Upon the binding of its substrate, the conformation of binding proteins changes, accompanied with hinge bending. This hinge bending behavior could be used to transduce the binding event into a fluorescent signal by using nanomaterials and nanostructures. Taking advantage of recombinant protein engineering techniques, various glucose-binding proteins could be designed and produced as versatile nanobiotools for glucose sensing. 

The glucose signal can be detected by the enzymatic catalytic reaction products of glucose oxidization. The glucose oxidization reaction is accompanied by the production of gluconic acid and $H_{2}O_{2}$. By correlating the amount of product produced with the fluorescent systems changes, the glucose concentration can be determined. Therefore, fluorescent nanomaterials for sensing pH changes and/or hydrogen peroxide are ready for glucose nanobiosensing. 

### 3.3. Glucose Fluorescent Nanobiosensors

**Figure 3:**
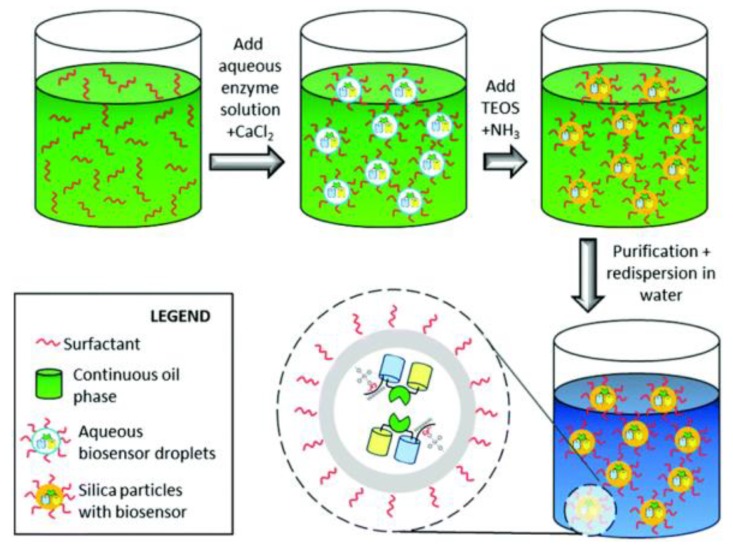
Schematic illustration of the process for protein encapsulation in silica nanoparticles. After the formation of a microemulsion, silica nanoparticles are formed by the addition of ammonium hydroxide to increase the pH. In the last step, the inverse microemulsion is redispersed in water to give an aqueous silica dispersion with the fluorescence resonance energy transfer (FRET)-based biosensor encapsulated in the silica nanomatrix. A specific interaction between the silica matrix and the biosensor is mediated by a silica-calcium-hexa-histidine-tag complex. Reproduced from Reference [102] with the permission of the Royal Society of Chemistry.

A fluorescence resonance energy transfer (FRET)-based glucose biosensor stabilized by encapsulation in silica nanoparticles was prepared (Figure 3) [102]. As demonstrated in the encapsulation process shown in Figure 3, a microemulsion fabrication method was adopted in the synthesis. The silica matrix shields the FRET biosensor from thermal and chemical denaturation. A specific interaction between the hex-histidine-tag of the biosensor protein and a calcium silica complex was achieved, which preserved its affinity to glucose. This study revealed that the silica matrix has a stabilization effect on biomolecules compared to bare FRET-biosensors. This silica-coated fluorescent glucose nanobiosensor is very promising for in vitro and in vivo glucose sensing. 

**Figure 4:**
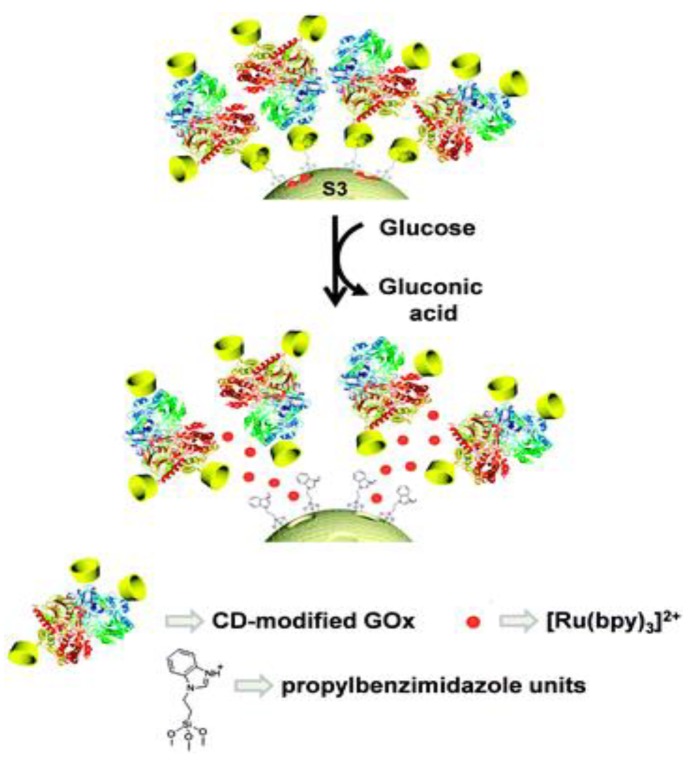
Ru(bipy)_3_^2+^-loaded mesoporous silica capped with cyclodextrin-modified glucose oxidase (CD-GOx) for the detection of glucose. Reproduced from Reference [30] with the permission of the Royal Society of Chemistry.

A gated mesoporous silica nanobiosensor for glucose sensing was prepared (Figure 4) [30]. The fluorescent reporter (ruthenium bipyridine complexes) was loaded into the silica mesopores. The mesopores outlets were grafted with propylbenzimidazole moieties. Then the cyclodextrins-modified glucose oxidase (CD-GOx) bioconjugates interacted with propylbenzimidazole groups by forming an inclusion complex, which blocked the loaded fluorescent reporters. After glucose was introduced, gluconic acid was produced, causing the protonation of the benzimidazole group. Then the CD-GOx bioconjugates were detached from the silica nanoparticles, triggering the release of fluorescent ruthenium bipyridine complexes. A linear glucose responsive range of 0.1 mM–10 mM was achieved by this nanobiosensor assay. Blood glucose levels of healthy individuals and diabetics are 3 mM–8 mM and 9 mM–40 mM, respectively. Therefore, gated mesoporous silica nanobiosensors are promising for the design of fluorescent nanobiosensors for glucose detection. 

**Figure 5:**
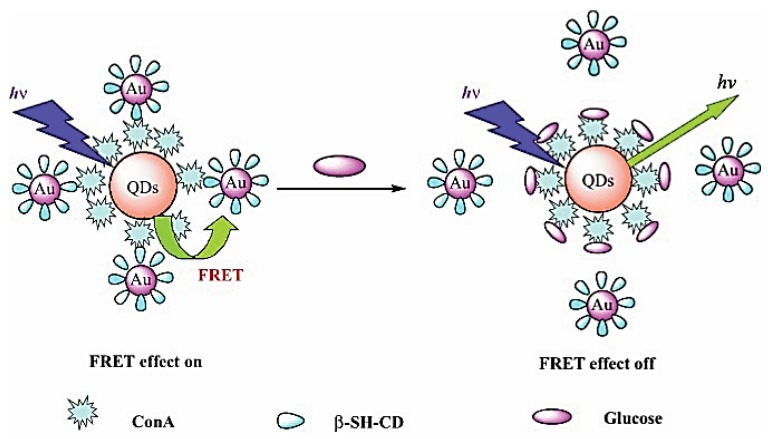
Illustration of the glucose sensor made of QDs, ConA, and Au NPs. Reproduced from Reference [103] with the permission of John Wiley and Sons.

A competitive fluorescent glucose nanobiosensor was constructed as shown in Figure 5 [103]. The design consisted of modified quantum dots (QDs) and plasmonic gold nanoparticles (Au NPs). The sensing mechanism is achieved through FRET between QDs (energy donor) and Au NPss (energy quenching acceptor). The surface of QDs is modified with Con A; meanwhile, β-SH-cyclodextrin is used to conjugate Con A onto the surface of the gold nanoparticles. Before glucose is introduced, the QDs and Au NPs are connected to Con A, and the fluorescence of the QDs is quenched by the plasmonic Au NPs. Once glucose is introduced, the glucose can compete with β-SH-cyclodextrin to bind onto Con A, therefore, the plasmonic Au NPs and QDs are staying apart. As a result, the emission of QDs will be recovered. Experimental results demonstrated that an increase in fluorescence intensity is proportional to glucose concentration in the range of 0.1–50 µM when optimized experimental conditions are reached. Also, the serum test results showed that this fluorescent glucose nanobiosensor has excellent glucose selectivity over other sugars and most biological interferents. 

**Figure 6:**
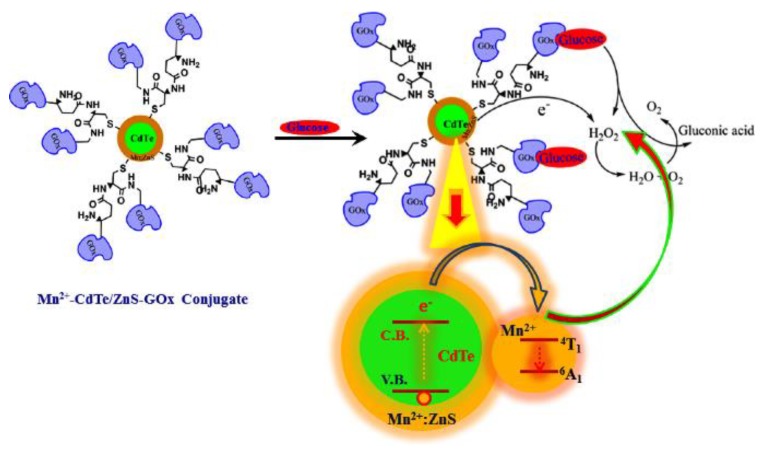
Schematic representation of the glucose sensor made of glucose oxidase-modified quantum dots. Reprinted from Reference [104], Copyright (2017), with permission from Elsevier.

A glucose oxidase-modified quantum dots-based glucose fluorescent nanobiosensor was developed [104]. As shown in Figure 6, the Mn-doped CdTe/ZnS quantum dots were modified with glucose oxidase on the surface. When glucose molecules were introduced into the nanobiosensor system, the glucose was oxidized to produce hydrogen peroxide and gluconic acid. The hydrogen peroxide then quenched the quantum dots fluorescence; thus, the glucose concentration was correlated to the fluorescence of the quantum dots. The experimental results showed that the fluorescence this nanobiosensor was proportional to the glucose concentration in the range of 0.1 nM–10 μM. 

**Figure 7:**
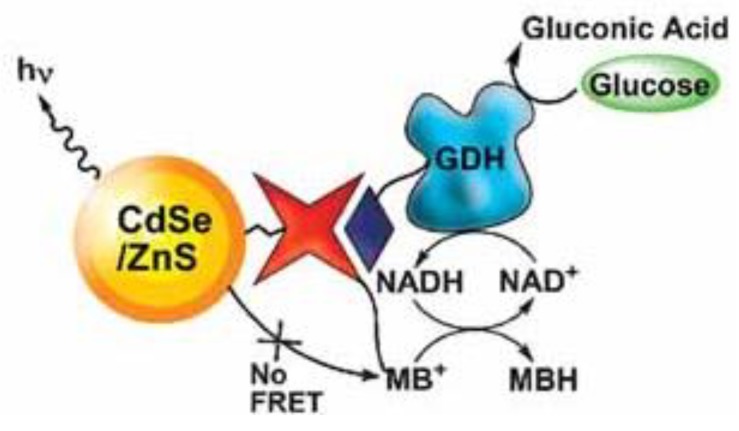
The glucose dehydrogenase biocatalyzed generation of NADH by the oxidation of glucose enables the fluorescence detection of glucose by methylene blue-functionalized CdSe/ZnS quantum dots. Reproduced from Reference [105] with the permission of John Wiley and Sons.

Another quantum dots-based glucose nanobiosensor was developed utilizing glucose catalytic reactions (Figure 7) [105]. Glucose dehydrogenase-modified CdSe/ZnS quantum dots were prepared to sense glucose. The sensing technique employed was FRET. The nanobiosensing mechanism is demonstrated in Figure 7. Before adding glucose, the fluorescence of the quantum dots was quenched by methylene blue. After adding glucose, biocatalytic reactions took place and the fluorescence of the quantum dots was recovered. In the biocatalytic reaction, glucose was converted to gluconic acid, and the glucose dehydrogenase biocatalytic reaction was mediated by the NADH−NAD^+^ pair, in which methylene blue was reduced by NADH to colorless MBH. Then the fluorescence resonance energy transfer between quantum dots and methylene blue was terminated. The detection limit of this fluorescent glucose nanobiosensor was as low as 10^−5^ M. 

**Figure 8:**
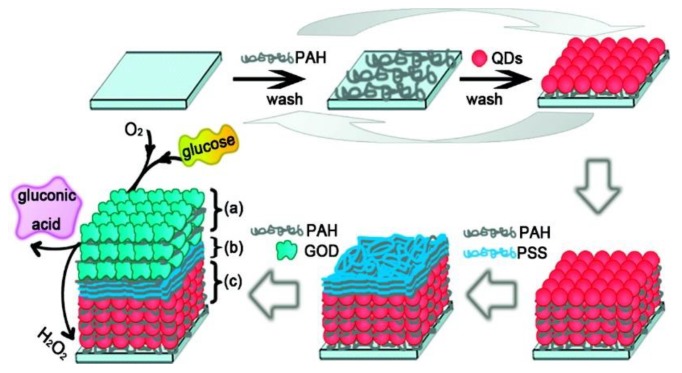
Sensing assembly: (a) top three bilayers of PAH/GOD, (b) three bilayers of PAH/PSS, and (c) 12 bilayers of PAH/CdTe QDs. PAH = poly(allylamine hydrochloride), GOD = glucose oxidase, PSS = polystyrenesulfonate, QD = quantum dots. Reprinted with permission from Reference [106], Copyright (2009) American Chemical Society.

A fluorescent nanostructured glucose biosensor chip utilizing glucose oxidase was developed by Tang et al. (Figure 8) [106], in which a layer-by-layer assembly technique was used to fabricate the thin film sensor. Firstly, polymer poly(allylamine hydrochloride) (PAH) and CdTe quantum dots were assembled on a substrate. Then, three bilayers of polymer film of poly(allylamine hydrochloride) (PAH) and polystyrenesulfonate (PSS) were assembled on top to avoid possible interference. Next, glucose oxidase (GOD or GOx) and PAH were deposited on top of the film. The layer-by-layer thin film structure of (PAH/CdTe)_x_(PAH/PSS)_3_(PAH/GOD)_y_ was constructed. The fluorescence of CdTe quantum dots was quenched by the H_2_O_2_ produced by the glucose catalytic oxidization reaction. This glucose sensor has the advantage of adjustment of the quantum dots layer composition and the glucose oxidase layer compositions, allowing adaptation to different sensing environments. The experimental results showed that the glucose responsive range for (PAH/CdTe)_12_(PAH/PSS)_3_(PAH/GOD)_3_ was 0.5 mM–16 mM. 

Efforts towards fluorescent sensing tear glucose studies are discussed. Human tear glucose is in the range of 0.16±0.03 mM for non-diabetics and 0.37±0.05 mM for diabetics [107]. Although the relationship between tear glucose concentration and blood glucose concentration is not strongly correlated, investigations of tear glucose sensing provide insight on a non-invasive glucose fluorescent detection method for patients. As research continues to develop, studies on tear glucose sensing are paving the way to a better diagnostic approach for glucose sensing. 

**Figure 9:**
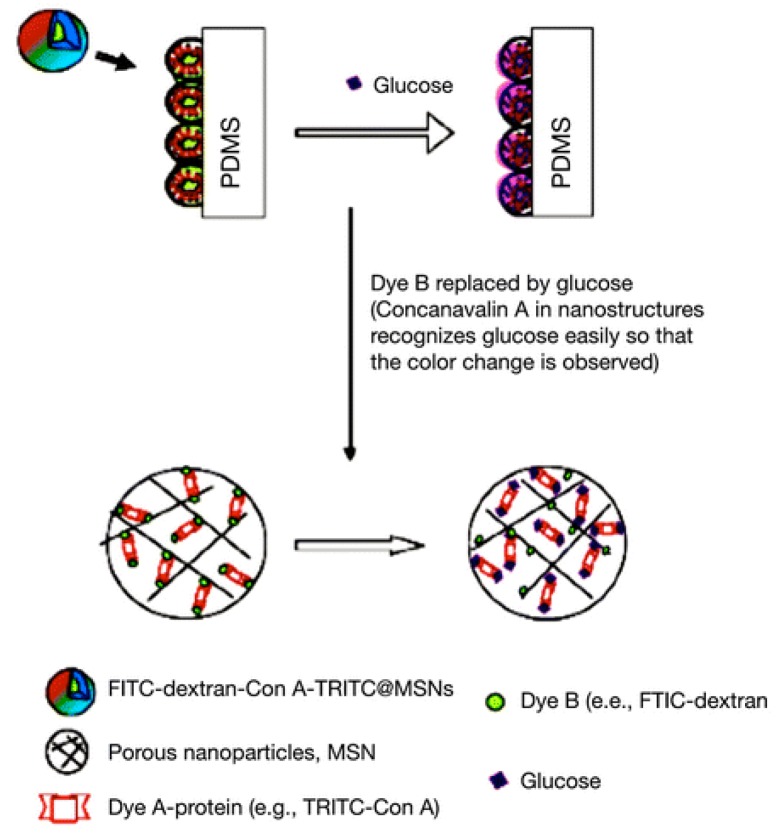
Steps for producing the nanostructured fluorescence resonance energy transfer nanobiosensor for monitoring tear glucose. Reprinted with permission from Reference [108]. Copyright © [2013] (Jin Zhang). Reprinted with the permission of SAGE Publications.

A highly sensitive nanostructured fluorescent biosensor was developed [108,109]. The fluorescent nanobiosensors sensing mechanism is illustrated in Figure 9. Dextran-fluorescein isothiocyanate-conjugated silica nanoparticles (FITC-dextran-silica) interacted with tetramethylrhodamine isothiocyanate-labeled Concanavalin A (TRITC-Con A) by Concanavalin A binding to the dextran molecules. Fluorescence resonance energy transfer pairs between fluorescein isothiocyanate and tetramethylrhodamine isothiocyanate were formed (FITC-TRITC). Then these fluorescent nanomaterials were deposited onto the poly(dimethylsiloxane) (PDMS) surface in the format of a fluorescent chip device for glucose nanobiosensing. The introduction of glucose replaced the TRITC-Con A from the chip, causing fluorescence resonance energy transfer (FITC-TRITC) ratio changes corresponding to glucose concentration. This fluorescent nanobiosensor chip can sense glucose in less than 2 min, and the glucose sensing range is 0.04 M–4 M. The durability of this chip was evaluated over five days. 

**Figure 10:**
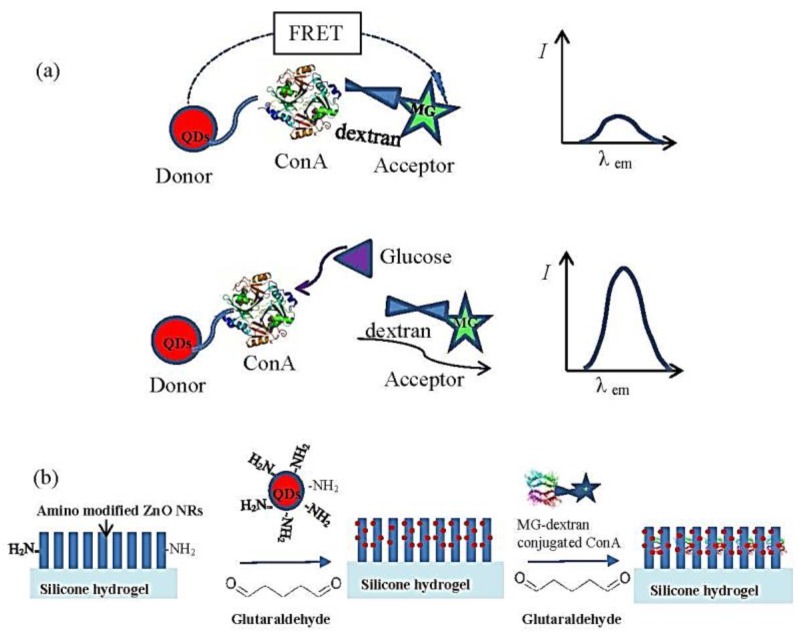
(**a**) Illustration of the FRET quenching sensor. (**b**) Immobilization of the nanostructured FRET quenching sensors on ZnO nanorod array deposited on silicone hydrogel. Reprinted from Reference [110], Copyright (2017), with permission from Elsevier.

Recently, Zhang group reported a new fluorescent glucose sensor based on the fluorescent patterned arrays as shown in Figure 10 [110]. This unique design can detect glucose level by both of fluorescence camera and fluorometer. Patterned ZnO nanorod arrays film coated on hydrogel was used as substrate, CdSe/ZnS QDs were conjugated onto the ZnO nanorods, and then Con A was conjugated onto the hybrid nanorods. Malachite green (MG) modified dextran was used as the fluorescence quenching molecule through binding onto Con A. When glucose was introduced, glucose competitively replaced dextran molecule and recovered the fluorescence of QDs. The experimental outcomes showed a glucose responsive range of 0.03 mM–3 mM, which covers the normal tear glucose concentrations of both healthy and diabetic people. 

**Figure 11:**
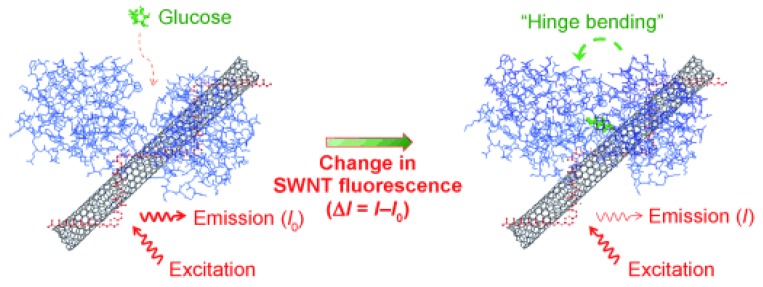
Glucose-binding protein (GBP) covalently conjugated to a fluorescent single-walled carbon nanotube (SWNT) is shown to act as an optical switch. Hinge bending response to glucose causes a reversible exciton quenching of the SWNT fluorescence with high selectivity. Reproduced from Reference [111] with the permission of John Wiley and Sons.

A glucose-binding protein/single-walled carbon nanotube nanobiosensor was developed (Figure 11) [111]. First, the carbon nanotube was wrapped and stabilized by carboxylated poly(vinyl alcohol) (cPVA). Then the glucose-binding protein (GBP) was covalently bioconjugated with the cPVA. The emission of the single-walled carbon nanotube was in the near-infrared area, which represents the biological transparent windows. The addition of glucose led to GBP hinge bending and conformation change. This conformation change influenced the near-infrared fluorescence of the carbon nanotubes. The experimental data showed that the glucose sensing range was 2.5 mM–50 mM and the fluorescence change was reversible due to the reversible GBP binding behavior. 

**Figure 12:**
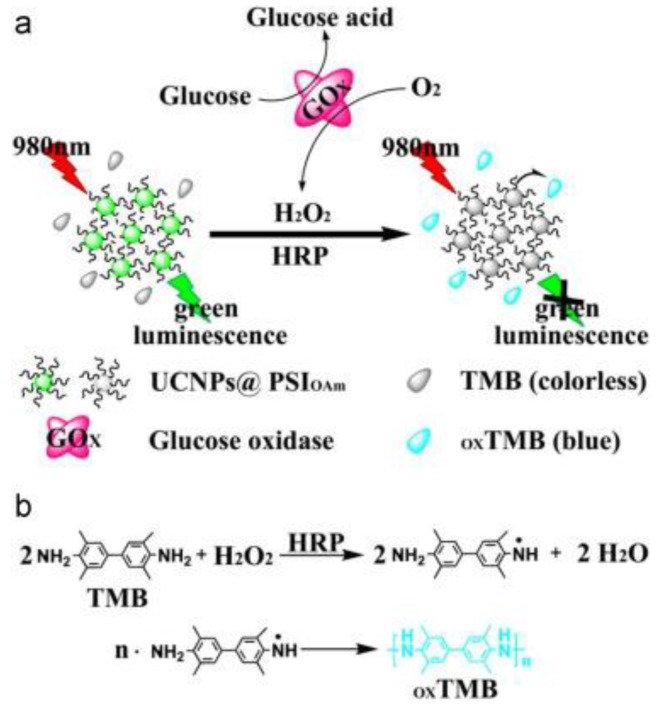
(**a**) Schematic diagram of the selective detection of H_2_O_2_ and glucose by using UCNPs. (**b**) The colorless TMB was oxidized into blue oxTMB by polymerization. Reprinted from Reference [112], Copyright (2015), with permission from Elsevier.

An upconverting nanomaterials-based fluorescent glucose nanobiosensor was studied, as shown in Figure 12 [112]. In the sensing assay, glucose was first incubated with glucose oxidase at 37 °C. This reaction produced hydrogen peroxide. The produced H_2_O_2_ was mixed with green-emitting NaYF_4_: Yb^3+^/Er^3+^ upconverting nanoparticles, colorless 3, 3’, 5, 5’-tetramethylbenzidine (TMB), and horseradish peroxidase (HRP). The mixture was further incubated at room temperature for 10 minutes before measuring the fluorescence. The sensing mechanism involves H_2_O_2_, which oxidized colorless TMB into blue oxidized TMB (oxTMB) with the assistance of HRP. As a result, the upconverting green emission was quenched by the oxidized TMB. The results showed that the glucose sensing linear range was 100 nM–4 µM and the analysis results of serum glucose samples were in good accordance with hospital detecting results. 

**Figure 13:**
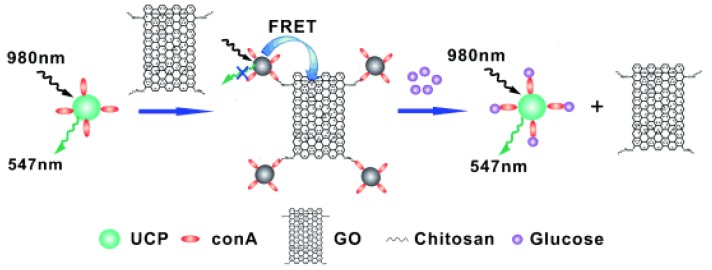
Schematic illustration of the UCP–GO biosensing platform and the mechanism of glucose determination. Reproduced from Reference [113] with the permission of the John Wiley and Sons.

Another glucose fluorescent nanobiosensor based on Concanavalin A upconversion-graphene oxide nanoconjugates was prepared for glucose sensing (Figure 13) [113]. The surface of upconverting nanoparticles was conjugated with Con A and the graphene oxide was modified with chitosan molecules. Then, the upconverting nanoparticles were bound to the chitosan-modified graphene oxide to form the nanobiosensor. Without glucose, the green emission of upconverting nanoparticles were quenched by graphene oxide, and after the glucose competitively bound Con A, the graphene oxide can be separated from the upconverting nanomaterials. As a result, FRET was terminated and the upconverting green fluorescence was restored. The analytical results showed that the glucose responsive range was 0.56 μM–2 μM. 

**Figure 14:**
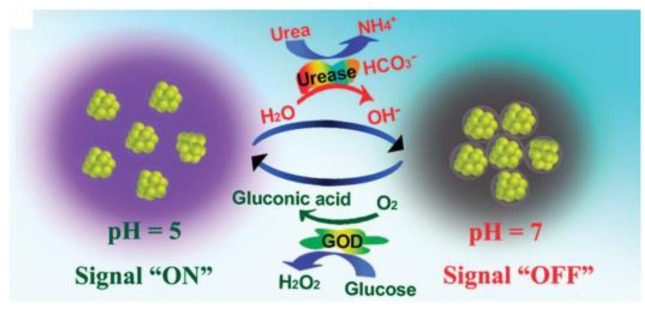
Schematic of the mechanism of the sensor for urea and glucose detection. Reproduced from Reference [114] with the permission of Nature Publishing Group.

Fluorescent silver nanocluster conjugates were developed as a nanobiosensor for glucose sensing (Figure 14) [114]. Silver nanoclusters were synthesized and its sensitivity to pH was used for glucose sensing. The pH sensitivity originated from the carboxylic groups (−COOH) on the silver nanocluster surface. These carboxylic groups can create an easily formed pH-sensitive molecular interaction (e.g. hydrogen bond) among silver nanoclusters. This interaction consequently affected the fluorescence of silver quantum clusters. Therefore, the gluconic acid produced by the glucose biocatalytic reaction could influence the fluorescence of the silver nanoclusters. The measured results indicated that the glucose sensing range was 0.3 mM–13 mM and the silver quantum clusters could be regenerated by recovering the pH (urea sensing, urea catalyzed by urease to increase the pH). 

**Figure 15:**
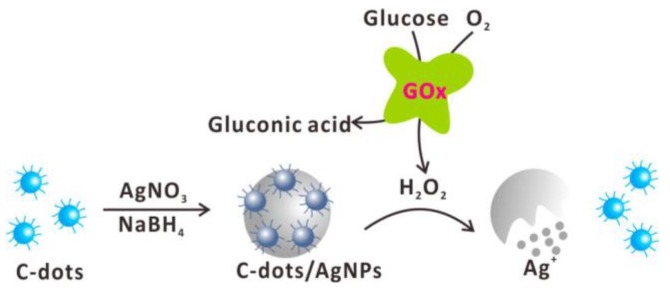
Fluorescence turn-on strategy for glucose detection based on a combination of carbon nanodots supported on silver nanoparticles and GOx-mediated oxidation of glucose. Reprinted with permission from Reference [115], Copyright (2016) American Chemical Society.

The advantage of carbon dots over other fluorescent nanomaterials is related to their water solubility and high biocompatibility after proper surface modification. Moreover, the surface of carbon dots often consists of carboxylic groups (−COOH), amine groups (−NH_2_), and other groups for further functionalization. A fluorescence turn-on strategy for glucose sensing was achieved by incorporating fluorescent carbon dots inside plasmonic silver nanoparticles, as shown in Figure 15 [115]. Fluorescent carbon dots were first encapsulated inside the plasmonic silver nanoparticles during the synthesis of silver nanoparticles. The glucose was oxidized by glucose oxidase to produced gluconic acid and H_2_O_2_. The H_2_O_2_-etched silver nanoparticles and the encapsulated carbon dots were consequently released. The advantage of this glucose nanobiosensor is the high biocompatibility of carbon dots and silver nanoparticles. The analytical results showed a linear response to glucose concentration in the range of 2 µM–10 µM. 

In conclusion, various fluorescent emitting/interacting nanomaterials are well suited for applications involving glucose biosensing. Fluorescent nanomaterials have been widely used as substrates for stabilizing biomoleculars to achieve high bioactivity, selectivity, and sensitivity. Nanomaterials including semiconductor quantum dots, mesoporous silica nanomaterials, upconverting nanomaterials, plasmonic gold/silver nanoparticles, fluorescent gold/silver quantum clusters, and the broad carbon nanomaterials of carbon dots, carbon nanotube, graphene/graphene oxide, and graphene quantum dots have been used to construct high-performance glucose fluorescent nanobiosensor assays and devices. 

Various glucose biological receptors, including Concanavalin A, glucose-binding proteins, glucose oxidase, glucose dehydrogenase, glucokinase, and hexokinase, are used in glucose nanobiosensing. Other assistant biomolecules like nicotinamide adenine dinucleotide (NAD-NADH) mediator and horseradish peroxidase (HRP) are also employed. Assistant saccharides such as dextran and chitosan are also involved in competition sensing assays.

## 4. Future Prospects and Challenges

Currently, the available glucometers on the market offer only invasive, non-real-time measures. There is a need to develop non-invasive, real-time, and highly efficient sensors for glucose detection. The conjugation of nanotechnology and fluorescence technology may provide an alternative solution. 

Biocompatibility and fluorescent properties of nanostructures have been improved with decades of effort. For example, the high quantum yield and photostability of semiconductor quantum dots make them attractive for continued research and development. However, the cytotoxicity of semiconductor quantum dots limits their clinical application in vivo. In order to improve the biocompatibility of QDs, a biocompatible coating layer is often applied to coat quantum dots before their further application in vivo and in vitro. Dye-doped silica nanomaterials have high biocompatibility. Moreover, silica nanomaterials can be modified with various chemical groups and methods of functionalization using universal silane chemistry. For example, Cornell dots with high brightness and biocompatibility demonstrate great potential for in vivo and in vitro applications. Clinical human trial and clinical translation reports indicate the good biocompatibility and non-cytotoxicity effect of Cornell dots [116,117]. Upconversion nanomaterials (UCNPs) are suitable for in vivo applications due to their high biocompatibility and unique upconverting fluorescence, which may minimize the damage of biological tissue. Plasmonic noble metal nanoparticles and noble metal quantum clusters show high biocompatibility. For instance, gold/silver nanoparticles are often used as fluorescent quenching components in a biosensor. On the other hand, carbon nanomaterials provide a broad range of nanotools for applications in glucose sensing. They have high biocompatibility as well as simple modification chemistry. Nanocarbon family members include graphene/graphene oxide, graphene quantum dots, carbon dots, single-walled/multi-walled carbon nanotubes, carbon nanodiamonds, and carbon nanohorns. They could be used as either fluorescent donors or fluorescence quenching acceptors in glucose sensor designs. 

Though the synthesis and surface modification of fluorescent nanomaterials have been well established in the laboratory, the mass production of nanostructures has not been fully conquered, representing a big hurdle in the development of nanostructured glucose biosensors with high sensitivity and excellent selectivity. With the development of new manufacturing processing including three-dimensional (3D) printing and nano/microscale assembly, nanostructured biosensors could be used in non-invasive and/or implanted devices to offer more convenient data acquisition models for continuous, user-friendly, and real-time glucose measures for the management of diabetes. For glucose sensing in food and pharmaceutical industries, the stability and durability in harsh environments like heat/cold, saline, and acidic/basic conditions need to be taken into consideration for the long-termed usage of nanostructured glucose biosensors. 

## Figures and Tables

**Table 1:** Summary of fluorescence-emitting/interacting nanomaterials

Fluorescence-Emitting/Interacting Nanomaterials	Pros	Cons
Semiconductor Quantum Dots [99,100]	Stable and strong fluorescence Multiplex sensing	Toxic elements like Cd UV excitation tissue auto-fluorescence
Fluorescent Silica Nanoparticles [101,102,103,104]	Facile surface modification, good carrier for biomedical applications	Matrix may contain unwanted structure directing agents or surfactants
Upconverting Nanoparticles [105,106]	980 nm excitation, low background noise Biocompatible Stable fluorescence	Fluorescence difficult to tune
Gold/Silver Nanoparticles/Nanoclusters [107,108,109,110,111]	Stable, bio-inert, nanoparticles with surface plasmonic properties, nanoclusters with fluorescence properties properties	Limited range of absorbance, nanoclusters instability concern
Fluorescent Carbon Nanomaterials [112,113,114]	Highly biocompatible Facile preparation by various methods Fluorescence strong/stable enough for biosensing Excitation-dependent emission, tunable by doping with nitrogen, etc. elements Possible upconverting fluorescence	Fluorescence mechanisms not fully understood Reproducibility
Graphene Nanomaterials [115,116,117]	Scale production, electrical and optical properties	Reproducibility, size control
